# A Novel Evaluation of World No Tobacco Day in Latin America

**DOI:** 10.2196/jmir.2148

**Published:** 2012-05-28

**Authors:** John W Ayers, Benjamin M Althouse, Jon-Patrick Allem, Daniel E Ford, Kurt M Ribisl, Joanna E Cohen

**Affiliations:** ^1^Children's Hospital Informatics Program in the Harvard-MIT Division of Health Sciences and TechnologyBoston, MAUnited States; ^2^Children's Hospital BostonBoston, MAUnited States; ^3^Harvard Medical SchoolBoston, MAUnited States; ^4^Johns Hopkins Bloomberg School of Public HealthBaltimore, MDUnited States; ^5^Keck School of MedicineUniversity of Southern CaliforniaLos Angeles, CAUnited States; ^6^Johns Hopkins School of MedicineBaltimore, MDUnited States; ^7^Gillings School of Global Public HealthUniversity of North CarolinaChapel Hill, NCUnited States; ^8^Lineberger Comprehensive Cancer CenterChapel Hill, NCUnited States

**Keywords:** Tobacco smoking, evaluation research, health communication, informatics, infoveillance, infodemiology

## Abstract

**Background:**

World No Tobacco Day (WNTD), commemorated annually on May 31, aims to inform the public about tobacco harms. Because tobacco control surveillance is usually annualized, the effectiveness of WNTD remains unexplored into its 25th year.

**Objective:**

To explore the potential of digital surveillance (infoveillance) to evaluate the impacts of WNTD on population awareness of and interest in cessation.

**Methods:**

Health-related news stories and Internet search queries were aggregated to form a continuous and real-time data stream. We monitored daily news coverage of and Internet search queries for cessation in seven Latin American nations from 2006 to 2011.

**Results:**

Cessation news coverage peaked around WNTD, typically increasing 71% (95% confidence interval [CI] 61–81), ranging from 61% in Mexico to 83% in Venezuela. Queries indicative of cessation interest peaked on WNTD, increasing 40% (95% CI 32–48), ranging from 24% in Colombia to 84% in Venezuela. A doubling in cessation news coverage was associated with approximately a 50% increase in cessation queries. To gain a practical perspective, we compared WNTD-related activity with New Year’s Day and several cigarette excise tax increases in Mexico. Cessation queries around WNTD were typically greater than New Year’s Day and approximated a 2.8% (95% CI –0.8 to 6.3) increase in cigarette excise taxes.

**Conclusions:**

This novel evaluation suggests WNTD had a significant impact on popular awareness (media trends) and individual interest (query trends) in smoking cessation. Because WNTD is constantly evolving, our work is also a model for real-time surveillance and potential improvement in WNTD and similar initiatives.

## Introduction

Tobacco is responsible for about 4 million premature deaths each year, mostly in developing countries [1] where smoking continues to find safe harbor [2]. Latin America has the seventh and third highest smoking-related mortality among men and women, respectively, in the 14 epidemiologic global regions [1]. Latin America’s share of smoking-attributable mortality will likely increase given the high prevalence of smoking: 43% of men and 23% of women smoke in Peru, 41% and 31% in Chile, 37% and 12% in Mexico, and 34% and 24% in Argentina, compared with 23% and 18% in the United States [3,4].

Mandated by the World Health Assembly in 1987 and commemorated annually on May 31, World No Tobacco Day (WNTD) aims to inform the public about the global tobacco epidemic using a combination of mass media approaches, including television, radio, and the Internet [5]. WNTD is believed to be especially important in regions with limited resources for cessation campaigns [6,7] and limited (albeit rapidly changing) tobacco control policy provisions, like in Latin America [8-11]. However, nearing the 25th anniversary of WTND, its effectiveness remains unexplored.

Because tobacco surveillance is typically annualized, it is exceedingly difficult to estimate the impact of a single awareness day [12]. In nearly all studies [13], investigators make comparisons using annual data from cross-sectional or cohort surveys before and after the intervention [14]; however, differences across the year may not be attributable to the intervention effect [15]. Sometimes investigators ask respondents in cross-sectional surveys to describe their exposure to an intervention, comparing those reporting exposure with those not reporting exposure [16]. Rarely, investigators use cross-sectional monthly survey responses to yield estimates on a finer temporal resolution [17], but these trends are costly to obtain and are still not useful for a single awareness day, given the primary effects are expected on a finer temporal resolution than months. Pioneering work in infodemiology and infoveillance provided a framework for how to assess WNTD’s potential effectiveness [18-23]. For instance, Twitter feeds presage cholera epidemics [24]; Internet search queries forecast dengue incidence [25]; and unstructured media reports provide the earliest alerts of disease outbreaks [26]. Building on this work, we employed a novel daily digital surveillance approach using freely available and public archives to estimate the effectiveness of WNTD in seven Latin American nations.

We monitored both intermediate and primary outcomes. First, WNTD may promote cessation awareness through the promotion of smoking cessation in news media [13]. By monitoring online news archives for articles promoting cessation, we captured cessation awareness trends that may have been motivated by WNTD. Second, WNTD may encourage smokers to search the Web for cessation resources [27]. By monitoring aggregate Internet search queries indicative of cessation, we captured interest in cessation that may have been motivated by WNTD. WNTD increases in cessation awareness may trigger increases in cessation interest; therefore, we examined the relationship between cessation news coverage and Internet search queries for cessation.

## Methods

To address the study objectives, we analyzed online news media promoting cessation and online search queries for cessation from 2006 to 2011 in Mexico, Colombia, Argentina, Peru, Venezuela, Chile, and Ecuador using a quasi-experimental design [15].

We monitored weekly news stories archived on Google News that mentioned quitting smoking (Google Inc, Mountain View, CA, USA; news.google.com). Google News captures a broad spectrum of print, radio, and television media, and therefore is a useful archive to monitor cessation media stories. To identify the country of origin for each article, we downloaded data from nation-specific Google News domains (eg, news.google.com.mx for Mexico). All stories that mentioned smoking cessation were monitored relative to all stories in each nation. The numerator was the number of articles containing the phrase “dejar de fumar” (literally “give up smoking”), chosen based on discussion with experts and native Spanish speakers familiar with linguistic nuances across the nations monitored. The denominator was the number of stories that contained any universal conjunctive phrases, such as those containing “de,” thereby indicating a count of *all *stories. We report the resulting quotient per 100,000 stories.

Aggregate query trends were downloaded from Google Insights for Search (www.google.com/insights/). We monitored all queries that included “dejar de fumar” by nation. Trends were analyzed on a relative search volume (RSV) scale, with queries normalized to the period with the highest search proportion—for example, RSV = 100 is the highest search proportion period (cessation queries versus all search queries), and RSV = 50 is 50% of the highest search proportion. This approach corrects for trending in absolute search volume that is usually increasing for all common queries [28].

We analyzed data on weekly and daily time trends. Visual inspection of the data suggested the likely impact of WNTD was a pulse effect, an immediate change in mean news or query trends, rather than a durable shift. Because WNTD is a global program, there are no nations that may serve as untreated controls. As a result, we specified interrupted time series [29] within each nation to estimate the mean RSV around and including WNTD (3 weeks, including the week of, the week before, and the week after WNTD) to capture the rise and decline in WNTD spikes in reference to a within-nation RSV sampling from the 12 weeks before and after the WNTD period. The weeks before and after WNTD were included to capture presaging and lingering increases in news and search that was visually evident in the data inspection. We estimated the effect size as a ratio between WNTD and reference periods to make estimates comparable across nations (Ho:(RSV_WNTD _– RSV_reference weeks_)/RSV_reference weeks _= 0) [30]. To find the amount of variance in search queries explained by media trends, we regressed news trends lagged by 0, 1, and 2 weeks against search terms and reported *r* *2 *for all periods and after selecting only periods around WNTD [31].

## Results

**Figure 1:**
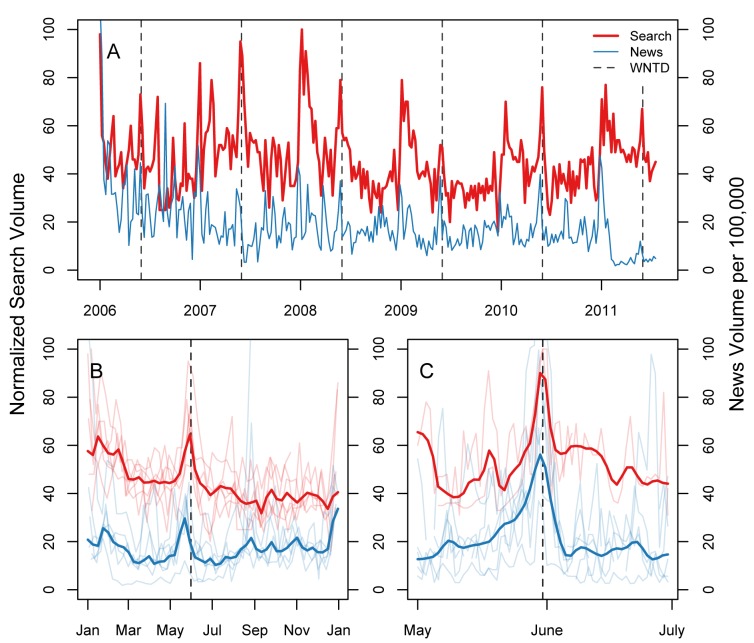
Example World No Tobacco Day (WNTD) time trends in Mexico, 2006-2011. (A) Entire series by week, (B) entire weekly series with mean annualized estimate, and (C) daily series with mean estimates. Daily query estimates were restricted to 2010 and 2011 due to data unavailability.

Figure 1 shows example cessation news coverage and cessation query trends in Mexico (patterns were similar in the six other study nations). Both news and query trends spiked in Mexico around WNTD. Averaging across years, cessation news trends typically peaked at 30 per 100,000 news stories the week of WNTD with cessation query trends peaking at 95% (RSV) of the highest search proportion week (with the highest RSV, 100, being on New Year’s Day 2008). Average trends across years (Figure 1B) suggested increases around WNTD were greater than the usual New Year’s spikes in Mexico. News and query peaks varied in their timing where there were strong buildups in news coverage beginning almost a month before WNTD, terminating 2 or 3 days after WNTD (Figure 1C), and queries had a pronounced peak around WNTD with queries remaining higher for a week after WNTD. Cessation news trends averaged 147 per 100,000 stories on WNTD, and queries had the highest search proportion day (RSV = 100) on WNTD.

**Figure 2:**
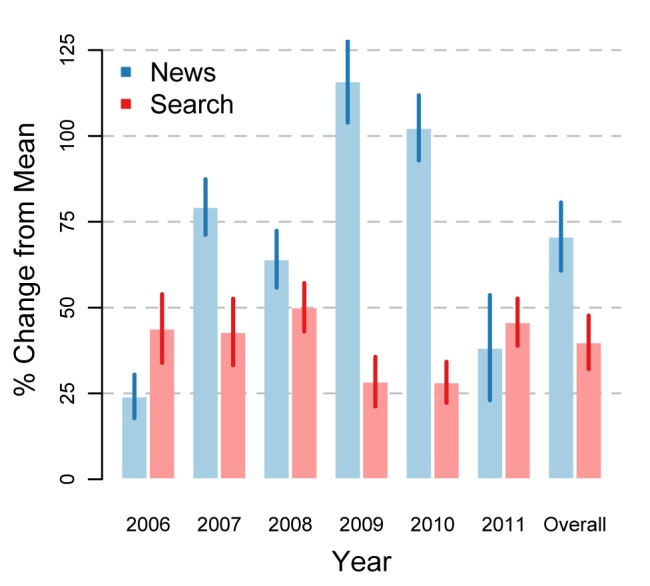
Pooled estimates of World No Tobacco Day effectiveness in Latin America, 2006-2011. Estimates are made comparing the week of (and before and after) World No Tobacco Day with 12 weeks before and after that period.

**Figure 3:**
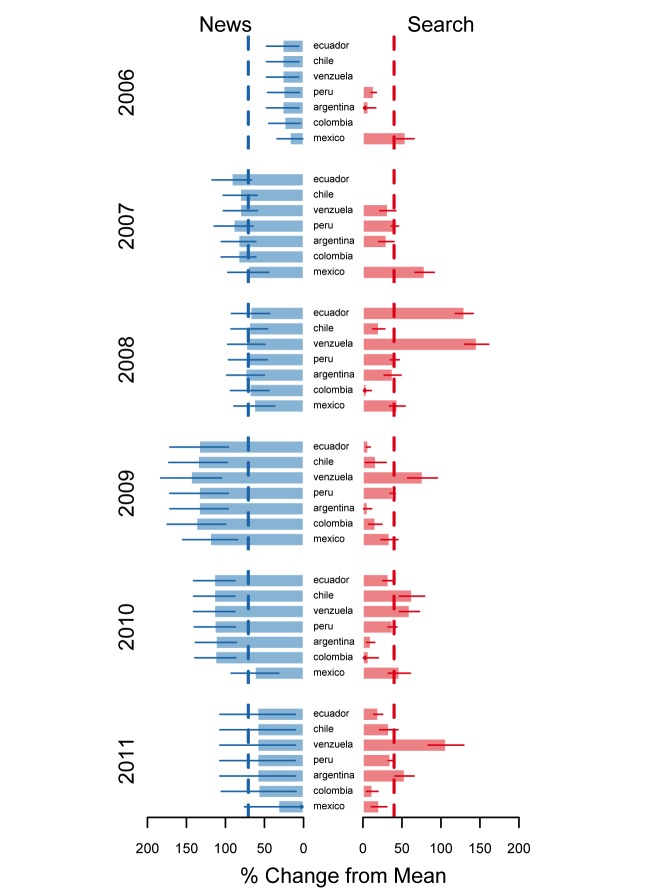
Nation-specific estimates of World No Tobacco Day effectiveness in Latin America, 2006-2011. Dotted line indicates average effect for all nations and years as a reference point. Data were unavailable in Ecuador, Chile, and Columbia for 2006 and 2007.

Statistical analysis suggested WNTD was associated with increases in cessation news coverage and cessation queries in all nations (Figure 2). Averaging across all nations and years suggested cessation news trends were 71% (95% confidence interval [CI] 61–81) higher and cessation query trends were 40% (95% CI 32–48) higher around WNTD than expected, as compared with trends 12 weeks before and after the WNTD period. Cessation news increases varied year to year, ranging from 24% (95% CI 18–30) to 116% (95% CI 103–128) for 2006 and 2009, respectively. Cessation query increases ranged from 28% (95% CI 21–36) to 50% (95% CI 43–57) for 2009 and 2008, respectively. Analyses by nation suggested news mentions of cessation ranged from 61% (95% CI 31–91) to 83% (95% CI 53–113) higher around WNTD in Mexico and Venezuela, respectively (Figure 3). Cessation queries ranged from 24% (95% CI 15–32) to 84% (95% CI 68–100) higher around WNTD in Argentina and Venezuela, respectively.

**Figure 4:**
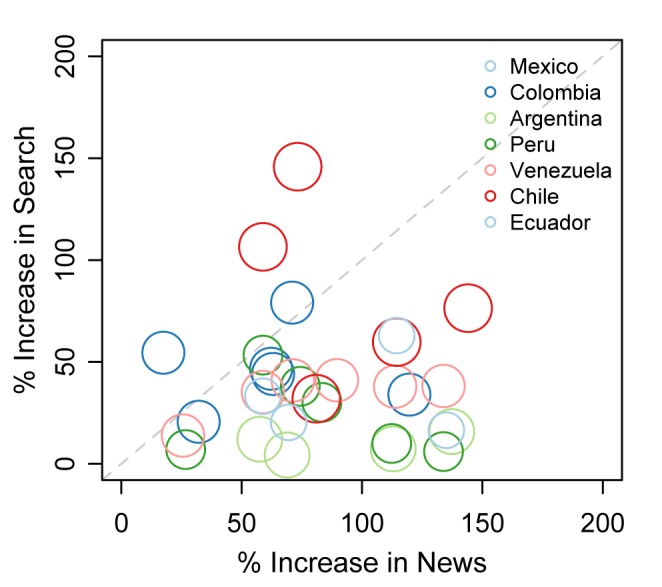
Cessation news coverage is positively associated with cessation Internet search queries. Nodes are sized according to mean annual search query volume, with nodes by nation and year.

Cessation news coverage explained limited amounts of variance in cessation queries. Multiple *r* *2 *ranged from .024 in Argentina to .516 in Mexico. Generally, cessation news coverage and cessation queries were not strongly associated across all time periods. However, the correlation between mean trends 3 months before to 3 months after WNTD was substantially higher (eg, *r *= .5 in Mexico). Figure 4 compares the magnitude of the effect sizes for search and news trend increases around WNTD by nation and year. Analyses showed increases in news trends (the week of, and before and after WNTD) were larger than increases in search query trends for nearly all the years, suggesting increases in cessation news coverage had diminishing returns on cessation queries during this narrow period. As such, a doubling in cessation news coverage corresponded with about a 50% increase in cessation queries.

Increases in cessation news coverage and cessation queries around WNTD need to be placed in practical perspective. Cigarette excise tax increases are the most effective tobacco control policy [32]. Around New Year’s Day in 2007, 2008, 2009, and 2011, Mexico increased its cigarette excise tax 4.7%, 1.3%, 1.2%, and 7.8%, respectively. We compared increases in cessation news coverage and cessation queries around WNTD with those from these tax years. In Mexico, increases in cessation queries around New Year’s Day (compared with the WNTD baseline) were as little as 10% (95% CI 2–23). WNTD-related increases were typically much larger than New Year’s-related increases in absence of a tax increase and were sometimes greater than increases when a new tax increase was also implemented. Regressing tax increases against WNTD increases across years and predicting the result for the overall mean suggested that WNTD increases in cessation news coverage and query trends approximated those for a 2.8% (95% CI –0.8 to 6.3) cigarette excise tax increase. Additionally, the estimate is based on a small sample (6 years), so while it is qualitatively accurate, the effect of WNTD may be equivalent to a somewhat lower or higher tax, especially since all tax increases occurred in addition to any New Year’s effects.

## Discussion

We demonstrated the feasibility of novel digital surveillance for tobacco control, yielding the first population estimates of WNTD’s effectiveness in the first program evaluation to use digital surveillance. WNTD coincided with large increases in cessation news coverage and cessation Internet search queries in seven Latin American nations. WNTD increases eclipsed New Year’s Day increases and approximated the effectiveness of the combination of New Year’s and a 2.8% cigarette tax hike. There was little correlation between news trends and query trends when comparing across the entire time series, but higher correlation when comparing trends around WNTD. This may indicate that news plays a minor role in increasing cessation queries generally but is magnified when the population is primed by WNTD [33].

This report builds on a programmatic agenda complementing tobacco control surveillance with digital data streams [34,35]. Epidemiologists have demonstrated the potential of digital surveillance for monitoring chickenpox [36,37], dengue [25,38], gastritis [36], influenza [18,39] including H1N1 [20], kidney stones [40,41], listeriosis [23], Lyme disease [42], methicillin-resistant *Staphylococcus aureus *[43], and salmonella outbreaks [22]. However, applications to health behaviors are very rare [20,27,34,35,44]. With smoking accounting for almost 6 million deaths each year [45], the application of digital surveillance to tobacco control may be of great significance. Herein we present our approach as a viable method to evaluate WNTD where traditional methods were ill equipped to do so.

While the infoveillance approach has many strengths, it is not without limitations. We only considered increase in news coverage around WNTD and did not consider the placement of news coverage (eg, lead versus minor story), but this may be captured by news volume, where increased volume is associated with premium placement. Cessation interest was only assessed for smokers with access to the Internet, but we assume that changes in cessation interest among those with access mirrors those without access [46]. Recent studies encourage this assumption, suggesting queries are valid proxies for a host of health outcomes, even in disparate regions [25,39]. For example, depression queries correlate with suicide rates [47] and cancer queries correlate with cancer-specific incidence rates [48]. We compared WNTD with cigarette excise taxes in Mexico (the only nation with several cigarette excise tax increases), and all tax increases occurred on New Year’s Day; hence, WNTD’s approximation to a 2.8% tax increase might be larger in other nations and when taxes do not increase during New Year’s.

Global health awareness campaigns are difficult to evaluate given sparse transnational data streams, and contemporary surveillance does not collect data on the same time dimensions as the campaigns that will likely effect change (eg, annual surveillance versus daily impacts) [12]. Tobacco control can learn much from adapting novel techniques that are gaining traction in biosurveillance [38]. Here we have built on validated approaches used in disease surveillance [25,38] and highlighted the potential advantages of these techniques over other tobacco control surveillance methods. Survey-based tobacco control surveillance has high financial costs, lacks timeliness, and may not reach some populations (eg, cell phone-only households) [49]. Also, responses gathered may be biased by strong social desirability [50]. Alternatives using passive data generation are only reliable in certain geographic areas and still require costly data processing (eg, nicotine replacement therapy sales) [51]. Digital surveillance, as such, is a critical forefront for tobacco control with real-time, public, and low-cost data streams useful for program and policy evaluations. Analyses of media and search query trends also afford greater transparency, as scientists may quickly replicate each other’s work, downloading data from regularly updated online archives. Extending our approach can serve as a continual evaluation system for global WNTD effects.

In part, these results may outline how well WNTD’s yearly themes resonate with Latin American populations. In 2009 and 2010 when WNTD focused on tobacco health warnings and smoking among women, respectively, cessation news coverage was the highest. News coverage increases were smallest when WNTD focused on general campaigns, such as promoting the World Health Organization Framework Convention on Tobacco Control (2011) and promoting awareness of the health effects of nonsmoking-related tobacco harms (2006). The variability in cessation search queries was much smaller year to year, but increases were generally larger during WNTD’s clean indoor air (2007) and restricting youth access to tobacco (2008) campaigns.

WNTD planners, and those responsible for tobacco cessation initiatives more broadly, may also use our results and techniques to scale up and refine the online components of their campaigns [52]. Since many search engines rank links according to the number of times searchers visit the page, or how many other pages link to a page, it is unrealistic to assume that creating cessation webpages, blogs, or YouTube videos will necessarily reach large numbers of tobacco users, even if they are seeking these resources. Search engine advertisements triggered by user-specific queries may be purchased for as little as US $0.01 per click. Advertised links appear on the first page of search results, ensuring that cessation queries meet webpages with current, health-conscious, and objective discussion of cessation when program planners advertise online. Search queries are a rare opportunity to reach smokers when they are thinking about cessation, while at the same time taking into account their stage in the cessation process with links individually tailored to optimize effectiveness [53,54]. By appropriately purchasing ad links, planners can ensure that evidence-based approaches maintain a strong first-page presence in cessation search results, especially around WNTD when many smokers appear interested in cessation. This is especially crucial given that most smokers attempt to quit without professional counseling or pharmacologic therapy [55].

The effectiveness of WNTD has been widely speculated [8], but to our knowledge these are the first empirical estimates of its impact. In evaluating this intervention, we highlight the importance of digital surveillance for performing difficult evaluations using real-time, low-cost, and transparent methods. Herein we find that WNTD is producing large increases in cessation news coverage and cessation Internet search queries, rivaling the effects of Mexico’s recent tobacco tax increases and New Year’s resolutions. Because WNTD is constantly evolving, our work is also a framework for real-time surveillance and potential improvement in WNTD.

## Abbreviations

CI: confidence interval
RSV: relative search volume
WNTD: World No Tobacco Day
